# Relaxor-Ferroelectric Films for Dielectric Tunable Applications: Effect of Film Thickness and Applied Electric Field

**DOI:** 10.3390/ma14216448

**Published:** 2021-10-27

**Authors:** Minh D. Nguyen, Doan T. Tran, Ha T. Dang, Chi T. Q. Nguyen, Guus Rijnders, Hung N. Vu

**Affiliations:** 1 MESA+ Institute for Nanotechnology, University of Twente, P.O. Box 217, 7500AE Enschede, The Netherlands; a.j.h.m.rijnders@utwente.nl; 2 International Training Institute for Materials Science (ITIMS), Hanoi University of Science and Technology, 1 Dai Co Viet Road, Hanoi 100000, Vietnam; doantranthi97@gmail.com (D.T.T.); danghaktck@gmail.com (H.T.D.); chintq@vnuf.edu.vn (C.T.Q.N.); 3 Mechanics and Civil Engineering, Vietnam National University of Forestry, Chuong My District, Hanoi 100000, Vietnam

**Keywords:** dielectric properties, tunability, figure-of-merit, relaxor ferroelectrics

## Abstract

The dielectric properties, tunability and figure-of-merit (*FOM*) of relaxor Pb_0.9_La_0.1_(Zr_0.52_Ti_0.48_)O_3_ (PLZT) films have been investigated. Dielectric measurements indicated that the dielectric constant (at zero-bias field), tunability and *FOM* are enhanced as the film thickness increases, which are mainly attributed to the presence of an interfacial layer near the film-electrode interface. Experimental results illustrated that a slight reduction is observed in both dielectric constant and tunability (−2%) in a wide-frequency range (10 kHz–1 MHz); meanwhile, the *FOM* value decreases significantly (−17%) with increasing frequency, arising from the higher dielectric loss value. The 1000-nm PLZT film shows the largest tunability of 94.6% at a maximum electric-field of 1450 kV/cm, while the highest *FOM* factor is 37.6 at 1000 kV/cm, due to the combination of medium tunability (88.7%) and low dielectric loss (0.0236). All these excellent results indicated that the relaxor PLZT films are promising candidates for specific applications in microwave devices.

## 1. Introduction

Tunable microwave devices are currently of high interest for applications as tunable dielectric filters, tunable delay lines and microwave phase shifts for steerable antennas [1,2,3,4,5]. In general, the tunable device can be a capacitor based on ferroelectric, ferromagnetic or a combination of ferroelectric/ferromagnetic materials. In these structures, an applied electric field can be used to alter the surface wave propagation by varying the dielectric constant of the ferroelectric layer, or by varying the permeability of the ferromagnetic layer by changing the applied magnetic field [3,6,7,8,9,10].

In ferroelectric-based tunable microwave devices, the high achievable tunability and low dielectric loss, corresponding to the large figure-of-merit (*FOM*) of the ferroelectric layer, are crucial parameters for their performance in practical applications. The tunability of a ferroelectric layer is determined by the following formula:

(1) $$T=\frac{\varepsilon_{r,0V}-\varepsilon_{E_{ap}}}{\varepsilon_{r,0V}}\times 100\%$$

where *ε_r_*_,0*V*_ is the dielectric constant at zero-bias field and *ε_E_ap__* is the dielectric constant a certain bias applied field (*E_ap_*).

The *FOM*, which is normally used to reflect the trade-off of dielectric materials for tunable devices [11,12], can be expressed as:

(2) $$FOM=\frac{\mathrm{Tunability}}{\tan\;\delta }$$

In Equation (2), the *FOM* factor is calculated considering the tunability and dielectric loss at a zero-bias field.

The tunability and *FOM* of ferroelectric films has been widely studied in materials including lead- and lead-free perovskites, in which the dependence of these properties on film properties, such as crystalline quality and thickness, were discussed. In general, tunability increases with film crystallinity and thickness [13,14]. Recently, multilayer films have also been investigated for improving tunability and *FOM* [11,15,16,17]. Yu indicated that the tunability and *FOM* values of multilayer *x*PbZr_0.52_Ti_0.48_O_3_/(1 − *x*)Bi_1.5_Zn_1.0_Nb_1.5_O_7_ (PZT/BZN) films can be changed by varying the PZT/BZN thickness ratio, and also by changing the number of layer in the structure [11]. Their results showed that the multilayer [PZT/BZN]*_N_*_=4_ structure with PZT/BZN thickness ratio of 3:1 had a large dielectric constant and low dielectric loss at zero bias field, due to the contribution of the interfacial layer near the PZT/BZN interface, resulting in the enhancement of tunability (~53.3%) and *FOM* (~65.6). The tunability and *FOM* values of lead-based, lead-free and multilayer films are summarized in Table 1.

The applied electric field (*E_ap_*) dependent tunability, calculated from Equation (1), indicates that the tunability increases with increasing maximum applied electric field (*E_ap,max_*), due to the decrease in *ε_E_ap,max__* (as will be described in the next section), resulting in the increase of (*ε_r_*_,0*V*_ − *ε_E_ap,max__*). In previous work, we have reported that the electric breakdown strength (*E_BD_*) of relaxor-ferroelectric Pb_0.9_La_0.1_(Zr_0.52_Ti_0.48_)O_3_ (PLZT) films was much higher than that of normal-ferroelectric Pb(Zr_0.52_Ti_0.48_)O_3_ (PZT) films [18]. More importantly, the *E_BD_* was also enhanced as the film thickness increased [19], which is contradictory to the inverse square root dependence of *E_BD_* on sample thickness (*t*, *E_BD_* ∝ *t*^−1/2^) [20,21,22,23]. However, this inverse relationship is considered valid when the thickness of samples is greater than the critical thickness (*t_critical_* ≈ 20 μm) [20,23,24]. This thickness-dependent breakdown strength regime is attributed to defects in the dielectric materials that can be introduced during fabrication process (extrinsic breakdown) [23]. Meanwhile, the thickness-independent breakdown strength of the dielectric materials was observed in thin sample ranges (lower than that of 200–500 nm, depending on the type of materials) [23]. In the PLZT films with a thickness range from 250 nm to 1000 nm, increase in the film thickness was found to enhance the breakdown strength, which was attributed to the reduction of space-charge injection as result in the decrease in leakage current with increasing film thickness [25,26].

**Table 1:** Tunability and *FOM* of lead-based and lead-free films.

Film	Thickness (nm)	*E_ap,max_* (kV/cm)	Frequency (kHz)	Tunability (%)	*FOM*	Refs.
PbZr_0.52_Ti_0.48_O_3_	1000	300	10	68.4	32.2	[27]
Pb_0.92_La_0.08_Zr_0.52_Ti_0.48_O_3_	690	300	10	68.9	19.1	[28]
Pb_0.92_La_0.08_Zr_0.52_Ti_0.48_O_3_	3000	1000	10	88.6	16.4	[29]
Pb_0.92_La_0.08_Zr_0.52_Ti_0.48_O_3_	3000	400	10	63.0	10.5	[30]
Pb_0.4_Sr_0.6_TiO_3_	500	300	1000	73.7	10.4	[31]
Pb(Zr_0.52_Ti_0.48_)O_3_/Bi_1.5_Zn_1.0_Nb_1.5_O_7_	800	500	100	53.3	65.6	[11]
BaSn_0.15_Ti_0.85_O_3_	420	80	100	70.0	17.0	[32]
Ba_0.6_Sr_0.4_TiO_3_	-	300	1000	72.2	24.6	[33]
0.06Nb(Zn_1/2_Ti_1/2_)O_3_-0.94Ba_0.7_Sr_0.3_TiO_3_	-	300	1000	37.1	43.2	[33]
Ba_0.45_Sr_0.55_TiO_3_	250	640	-	78.0	16.3	[34]
0.04Ba(Mg_1/3_Nb_2/3_)O_3_-0.96Ba_0.45_Sr_0.55_TiO_3_	250	640	-	72.0	24.0	[34]
Pb_0.9_La_0.1_Zr_0.52_Ti_0.48_O_3_	1000	800	1000	85.2	33.3	This study
Pb_0.9_La_0.1_Zr_0.52_Ti_0.48_O_3_	1000	1000	100	88.7	37.6	
Pb_0.9_La_0.1_Zr_0.52_Ti_0.48_O_3_	1000	1450	100	94.6	34.4	

In this study, we have investigated relaxor PLZT films with various film thicknesses in order to explore the correlation between applied electric field and dielectric, tunability and *FOM* properties. Moreover, the effect of applied frequency on the tunability and *FOM* of PLZT films has also been discussed in detail, as it determines the range of selectable frequencies for different microwave applications. The results in this study indicate that the relaxor-ferroelectric PLZT films with high breakdown strength demonstrate high tunability (~88.7%) and a large *FOM* (~37.6) at the high frequency of 100 kHz, making them a promising candidate for application in microwave tunable devices.

## 2. Experiment

Relaxor-ferroelectric Pb_0.9_La_0.1_(Zr_0.52_Ti_0.48_)O_3_ (PLZT) films with different thicknesses of 250, 500 and 1000 nm were deposited on 100 nm SrRuO_3_ (SRO) electrode-covered SrTiO_3_/Si 1-inch wafers, using pulsed laser deposition (PLD) method with a KrF excimer laser source (Lambda Physik, 248 nm wavelength). The deposition conditions of PLZT and SRO layers can be found in Ref. [19]. After deposition, the film capacitors with dimensions of 100 µm × 100 µm were patterned and structured by argon-ion beam etching for the SRO top-electrodes and by wet-chemical etching (HF/HCl solution) for the PLZT films.

The crystalline orientation and microstructure of the films were analyzed via X-ray diffraction θ-2θ scans (XRD), using a PANalytical X-ray diffractometer with Cu-K*α* radiation with a wavelength of 1.5405 Å, and also via cross-sectional scanning electron microscopy (SEM, Zeiss MERLIN). The polarization hysteresis (*P-E*) loops and switching current (*I_SW_-E*) curves were measured with the dynamic mode (DHM) of the TF-2000 Analyzer (aixACCT GmbH, Aachen, Germany). The capacitance-electric field (*C-E*) curves were done with the capacitance module of the TF-2000 Analyzer. A slowly sweeping dc-bias field with a small ac-field (10 kV/cm) and in the frequency range of 10 kHz to 1 MHz was applied during the capacitance measurement. The dielectric constant and dielectric loss were evaluated from the corresponding *C-E* curves.

## 3. Results and Discussion

**Figure 1:**
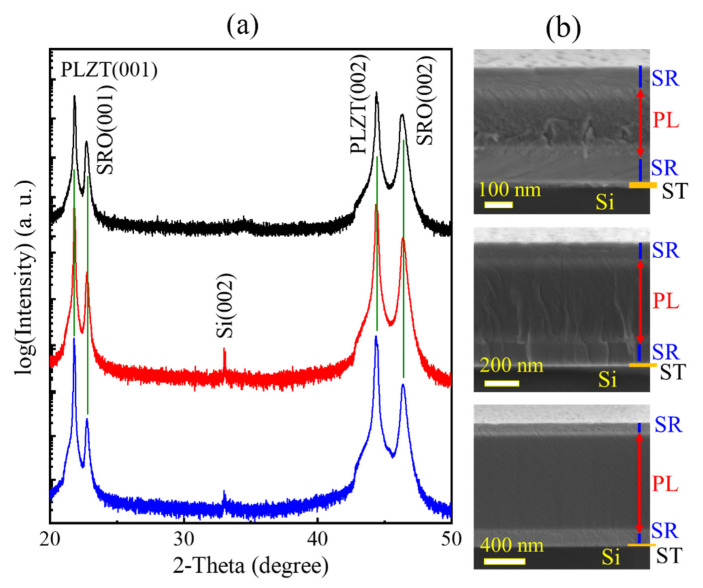
(**a**) XRD theta-2theta patterns and (**b**) corresponding cross-section SEM images of PLZT films.

Figure 1a shows the XRD θ-2θ scans of PLZT films, in which only the (00*l*)/(*l*00)-type diffraction peaks are observed. The out-of-plane *c*-lattice parameters, calculated from the position of the (002) peaks, are approximately 4.0796, 4.0806, and 4.0810 Å for 250, 500 and 1000 nm PLZT films, respectively. On the other hand, the (002) peak position is slightly shifted towards lower diffraction angles with increasing film thickness (see Figure S1a). Meanwhile, the in-plane *a*-lattice parameters, calculated from the position of the (200) peaks, are also slightly increased with increasing film thickness. The *a*-lattice parameters are approximately 4.0689, 4.0706, and 4.0716 Å for 250, 500 and 1000 nm PLZT films, respectively. In this study, the films are fully relaxed from the stress of the substrate because they are much thicker than the critical thickness (~80 nm) [35]. Figure S1c plots the *c*/*a* ratio as a function of film thickness, in which it decreases slightly from 1.0026 to 1.0023 as the film thickness increases from 250 to 1000 nm. The peak intensity of (002) and (200) (I(002)/I(200)) is also presented as a function of the film thickness in Figure S1c. The result shows that the greater the increase in the (002)/(200) ratio, the greater the degree of *c*-axis domain switching in the films. The microstructure of PLZT films can be analyzed using cross-sectional SEM measurement, illustrated in Figure 1b, in which all PLZT films show dense structure.

The polarization hysteresis (*P-E*) loops and corresponding switching current (*I_SW_*-*E*) curves of the PLZT films with various film thicknesses, measured to a maximum electric field of 800 kV/cm and 1 kHz, are shown in Figure S2a,b. Figure S2a shows that all the films exhibit slim *P*-*E* hysteresis loops with low remanent polarization (*P_r_* ≈ 2–3 μC/cm^2^) and small coercive field (*E_c_* ≈ 20 kV/cm). A slim *P-E* loop is one of the typical features of relaxor ferroelectric behavior [36,37,38], which is mainly attributed to the presence of polar nano-regions [39,40,41]. The slim *P*-*E* loop of relaxor materials can be maintained under high electric fields, which results in large energy storage density and high energy efficiency [37,42,43,44,45]. As shown in Figure S3a, moreover, the broad dielectric constant peaks with the shift of dielectric maxima towards a higher temperature with increasing frequency are observed in the PLZT films in this study, which is also another behavior characteristic of relaxors [42,46,47,48]. A similar result has also been observed for the sol-gel Pb_0.92_La_0.08_Zr_0.52_Ti_0.48_O_3_ films [49].

In contrast to normal ferroelectrics in that the *I_SW_*-*E* curves exhibit two sharp peaks of polarization switching at a high *E_c_* value, relaxor ferroelectrics show the rounded *I_SW_*-*E* curves with the switching peaks at a low *E_c_* value (near the zero field), due to the phase transition from ferroelectric macrodomains in normal ferroelectrics to polar nanoregions in relaxors [50,51,52]. In this study, the four peaks present in the *I_SW_*-*E* curves are observed in the relaxor PLZT films, in which the peak ‘I’ (Figure S2b) could be related to the electric-field-induced transition from a weak polar state (polar nanoregions) to a strong polar state, meanwhile the peak ‘II’ (Figure S2b) illustrates that the strong polar state returns back to relaxor state [53,54]. Moreover, the same value of switching peaks during loading (peak ‘I’) and unloading (peak ‘II’) the electric field process are observed in the *I_SW_*-*E* curves, which indicates that there is no irreversible electric-field-induced transition [53], on the other hand, no evidence of domain switching is observed in the PLZT films.

**Figure 2:**
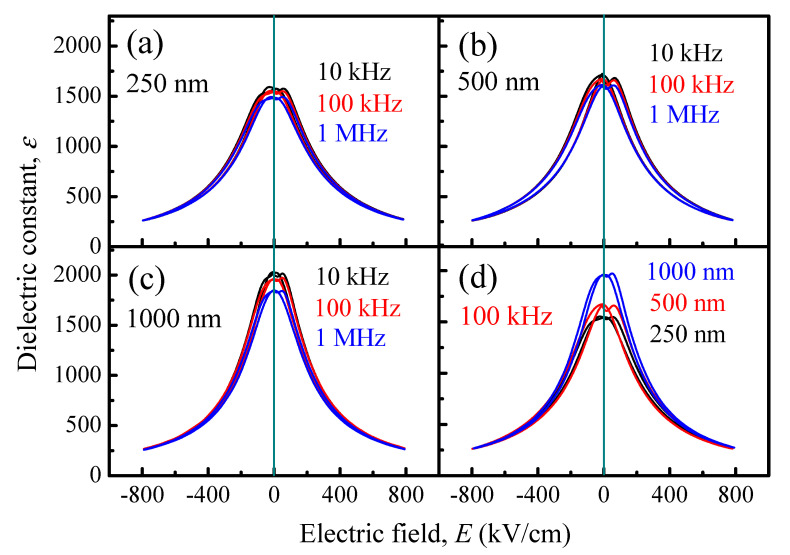
Dielectric constant–electric field (*ε*-*E*) curves of (**a**) 250-nm, (**b**) 500-nm and (**c**) 1000-nm PLZT films, as a function of frequency. (**d**) Comparison of *ε*-*E* curves of PLZT films measured at 100 kHz. The measurements were performed at 800 kV/cm.

The frequency dependent dielectric constant, dielectric loss, tunability and *FOM* of the relaxor-ferroelectric PLZT films have been investigated. Figure 2a–c illustrates the variation of the dielectric constant with electric field (*ε*-*E* curves) for 250, 500 and 1000 nm PLZT films, measured at a maximum applied electric field (*E_ap,max_*) of 800 kV/cm and at various frequencies. At zero bias, the dielectric constant values decreases with the increase in frequency (more information can be seen in Figure 4a and Figure S3b). Whereas, as can be seen in Figure 2a–c and Figure S3c, the dielectric constant saturates to a constant value at high electric-field region, regardless of frequency. It is well-known that the dielectric constant of dielectric materials is due to the contribution of two effects: intrinsic and extrinsic effects. The effect of intrinsic contribution on the dielectric constant is usually caused by deformation of the crystalline elemental cell. Meanwhile, the extrinsic contribution to dielectric constant is mainly governed by domain wall mobility and the interfacial layer near the electrode and the dielectric material.

In general, the decrease in the dielectric constant with increase in frequency could be attributed to the contribution of various effects, such as electronic polarization (occurs at frequencies up to 10^13^ kHz), ionic polarization (up to 10^10^ kHz), dipolar polarization (up to 10^7^ kHz) and space-charge polarization (up to 10^2^ kHz) [55,56]. Higher values of dielectric constant at low frequency are due to the contribution of all the various types of polarizations in the capacitors. In the present case, therefore, it is clear that the decrease in the dielectric constant of PLZT films at higher frequencies is due to the reduction of space-charge polarization effect. At lower frequencies, higher values of dielectric constant are associated with the accumulation of space-charge polarization at the grain boundaries [57,58], which can be explained using the Maxwell-Wagner polarization effect, under the assumption that the grains in the sample are separated by the insulating inter-grain barriers (leading to a formation of the ‘boundary-layer capacitor’ within the sample) [59,60,61]. The dielectric constant (*ε_r_*) of a ferroelectric material due to the motion of domain walls under various frequencies (*f*) can be described in terms of the Rayleigh model [62,63,64,65]:

(3) $$\varepsilon_{r}=\varepsilon_{r}\left(f\right)+\alpha_{e}\left(f\right)E_{ap,max}$$

(4) $$\varepsilon_{r}\left(f\right)=\varepsilon_{r,initial}-e\ln\left(f\right)$$

(5) $$\alpha_{e}\left(f\right)=\alpha_{e,initial}-a\ln\left(f\right)$$

where *ε_r,initial_*, *α_e,initial_* and *E_ap,max_* are the reversible dielectric constant at zero electric field, the irreversible Rayleigh parameter and the applied electric field, respectively. The magnitude of *α_e_E_ap_* represents the contribution of irreversible domain walls to dielectric properties. *e* and *a* are the frequency dependent dielectric constant and the Rayleigh parameters, respectively. Figure S4a shows the linear decrease of dielectric constant at zero field with the logarithm of the frequency. Therefore, it can be concluded that the extrinsic contribution to the dielectric constant of PLZT films is mainly attributed to domain wall motions, as similar to the case of ferroelectric films [64,66].

In the high-field region, the dielectric constant decreases as the driving electric field increases beyond the *E_c_* value (*E_c_*—the field at the peaks of *ε*-*E* curve, where the *ε* value is higher associated with high density of domain-wall motion and domain switching), as most switchable domains are aligned in the same direction of DC bias field, and also as the disappearance of the domain wall occurs. Therefore, the dielectric constant is small, as it is mainly associated with the variation of dipoles (hereafter known as the intrinsic contribution) [66,67]. Figure S3c indicates that the forward and backward curves of *ε*-*E* for PLZT films, measured at 800 kV/cm and various frequencies, are nearly overlapping in the high-field regions (−500 kV/cm to −800 kV/cm). In other words, the *ε*-*E* curves of PLZT films are independent of measured frequency in the high-field regions.

To investigate the film thickness dependence of the dielectric constant, the *ε*-*E* curves of PLZT films with film thickness from 250–1000 nm, measured at 800 kV/cm and 100 kHz, are compared in Figure 2d. The results indicate that the dielectric constant at zero electric field (*ε_r_*_,0*V*_) increases with increasing film thickness (see also in Figure S4b), and moreover the dielectric constant is near-constant at higher electric fields (Figure S3c,d). Previous reports indicated that a reduction in dielectric constant in ferroelectric films can be explained by the formation of a thin layer with lower dielectric constant, due to the concentration of space charge carrier as oxygen vacancies and/or imperfection induced during film growth, near the film/electrode interfaces [14,32,66,68].

If *C_P_* is the capacitance due to the PLZT film and *C_i_* is the interfacial capacitance between film and electrode, the effective (measured) capacitance (*C_eff_*) can be given as [69,70]:

(6) $$C_{eff}=\frac{C_{P}C_{i}}{C_{P}+C_{i}}$$

Equation (6) can be then expresses as follow:

(7) $$\frac{A}{C_{eff}}=\frac{A}{C_{i}}+\frac{A}{C_{P}}=\frac{t_{i}}{\varepsilon_{i}\varepsilon_{0}}+\frac{t-t_{i}}{\varepsilon_{P}\varepsilon_{0}}$$

where *A* is the surface area of top-electrode, and *t* and *t_i_* are the total film thickness and the interfacial layer thickness, respectively. *ε_P_* and *ε_i_* are the dielectric constant of PLZT and interfacial layers, respectively. *ε*_0_ (=8.854 × 10^−12^ F/m) is the dielectric constant of free space.

If *C_P_* << *C_i_*, or in other words, the thickness of the PLZT film is much greater than of the the interfacial layer (*t_P_* = *t* − *t_i_* >> *t*_i_), *C_eff_* will be equal to *C_P_*. Therefore, at higher film thicknesses, *C_eff_* will tend towards the actual dielectric constant of the PLZT film. Figure S4b shows the *A*/*C* ratio as a function of film thickness, in which the experimental data can be fitted by a linear relationship between the *A/C_eff_* and the film thickness. It can be found that *t_i_*/*ε_i_* ≈ 0.046 nm, which indicates the presence of the interfacial layer at the PLZT/SRO interface [66,71,72].

**Figure 3:**
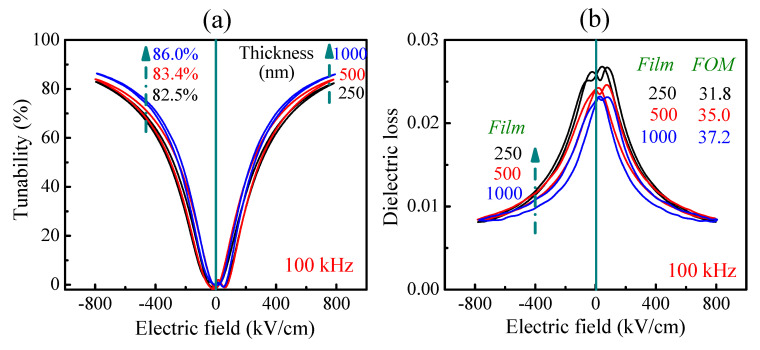
(**a**) Tunability curves which were calculated from the corresponding dielectric constant curves (Figure 2d), and (**b**) dielectric loss curves, of PLZT films as a function of film thickness measured at 800 kV/cm and 100 kHz.

The tunability of PLZT films’ dependence on electric bias field are calculated from Figure 2d and displayed in Figure 3a. According to Equation (1), tunability is mainly dependent upon the dielectric constants at zero bias field (*ε_r_*_,0*V*_) and at maximum applied electric field (*ε_E_ap,max__*). Under *E_ap,max_* of 800 kV/cm and 100 kHz, the tunability of 250, 500 and 1000 nm PLZT films is about 82.5, 83.4 and 86.0%, respectively. On the other hand, the increasing film thickness results in a significant enhancement in tunability, due to an increase in both *ε_r_*_,0*V*_ and (*ε_r_*_,0*V*_ − *ε_E_ap,max__*) values, as discussed above. From the combination of tunability (Figure 3a) and dielectric loss (tan *δ*, at zero bias field, Figure 3b), the values of the corresponding *FOM* values are calculated as 31.8, 35.0 and 37.2, for 250 nm, 500 nm and 1000 nm–thick PLZT films, respectively.

**Figure 4:**
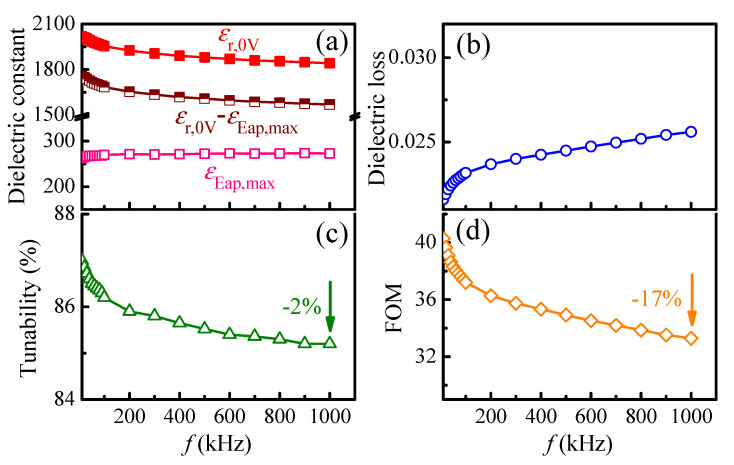
Operating frequency dependent (**a**) dielectric constant at zero field (*ε_r,0V_*) and at certain bias field (*ε_E_ap__*), (**b**) dielectric loss at zero field (tan *δ*), (**c**) tunability (*T*) and (**d**) figure-of-merit (*FOM*), of 1000-nm PLZT films. The measurements were done at 800 kV/cm.

Figure 4 shows the dielectric, tunability and *FOM* properties of the 1000 nm PLZT films, measured at 800 kV/cm, as a function of operating frequency. The dielectric constant at a certain field (*ε_E_ap__*) is near-independent of frequency, and then *ε_r_*_,0*V*_ and (*ε_r_*_,0*V*_ − *ε_E_ap,max__*) values show a similar trend, in which they decrease with increasing frequency (Figure 4a). Therefore, the tunability (as a ratio of (*ε_r_*_,0*V*_ − *ε_E_ap,max__*) and *ε_r_*_,0*V*_ values) is also slightly reduced. The decrease in *ε_r_*_,0*V*_ value and tunability at high frequencies are related to free dipoles oscillating in an alternative applied electric field (such as the damped motions of dipole oscillators), due to the reduction in relaxation time (*τ* = 1/frequency) [9,73,74]. Moreover, due to the increase in dielectric loss with increasing frequency (Figure 4b), it is found that the reduction rate of the *FOM* is much higher than that of tunability (Figure S5b). The reduction of tunability and *FOM* is about −2% and −17%, respectively, with the increase in frequency from 10 kHz to 1 MHz (Figure 4c,d).

**Figure 5:**
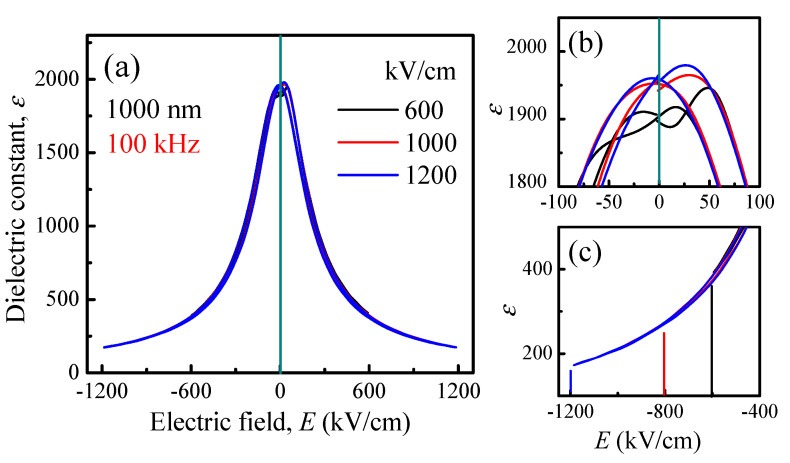
(**a**) Electric field dependent *ε*-*E* curves of 1000-nm PLZT films measure at 100 kHz. Zoom-in (**b**) near the peaks and (**c**) in the high-field regions of *ε*-*E* curves.

The maximum applied electric field (*E_ap,max_*) dependence of the *ε*-*E* curves of 1000 nm PLZT films measured at 100 kHz is shown in Figure 5a. In general, the relationship between dielectric constant and applied electric field can be described by Johnson’s formula [75,76]:

(8) $$\varepsilon_{E}=\frac{\varepsilon_{r,0V}}{{\left(1+\alpha \varepsilon_{r,0V}E^{2}\right)}^{1/3}}$$

where *α* is a positive constant. This equation is valid in cases wherein the value of *E* is larger than that of the *E_c_* value.

**Figure 6:**
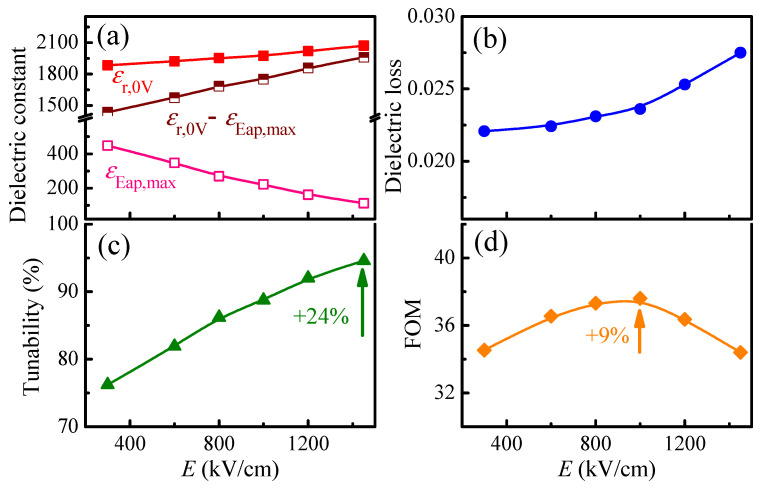
Applied electric-field dependent (**a**) dielectric constant at zero field (*ε_r_*_,0*V*_) and at certain bias field (*ε_E_ap,max__*), (**b**) dielectric loss at zero field (tan *δ*), (**c**) tunability (*T*) and (**d**) figure-of-merit (*FOM*), of 1000-nm PLZT films. The measurements were done at a frequency of 100 kHz.

Equation (8) shows that the *ε_r_*_,0*V*_ value of a dielectric material is independent of maximum applied electric field (*E_ap,max_*), and that dielectric constant decreases with increasing electric field (in the large-field region, *E* > *E_c_*). Figure 5c illustrates that the *ε*-*E* curves (both forward and backward) coincide with each other in the higher-field regions, following the relationship as indicated in Equation (8); this means that the dielectric constant value at *E_ap,max_* decreases with increasing value of *E_ap,max_* (Figure 6a). A magnification of the low-field region in Figure 5a, presented in Figure 5b, shows that the *ε_r_*_,0*V*_ value slightly increases with higher *E_ap,max_* (see also in Figure 6a). The difference between calculated and experimental results for the *ε_r_*_,0*V*_ value, in this case, can be explained by the existence of the polar nanoregions in the relaxor PLZT films. Cheng et al. indicated that there is a wide-range of the size distribution of the polar nanoregions, in which the polar nanoregions can change their size by applying electric field [77]. With the increase in applied electric field, the total dipolar moment per unit volume increases due to the increase in the dimension of polar nanoregions in some directions, but it decreases in other directions, thus resulting in an enhancement in *ε_r_*_,0*V*_ value [77]. For comparison, the *ε*-*E* curves of normal-ferroelectric Pb(Zr_0.52_Ti_0.48_)O_3_ films have been measured and are shown in Figure S6. This result indicates that the dielectric constants of ferroelectric materials defined at zero-bias field and in the high-field region are independent of *E_ap,max_*.

The *E_ap,max_*-dependent dielectric constant and dielectric loss of 1000 nm PLZT films are presented in Figure 6a,b. Similar to that of electric field-dependent dielectric constant, the tunability increases with an increase in *E_ap,max_*, which can be explained by the faster enhancement rate of the difference between the *ε_r_*_,0*V*_ and *ε_E_ap,max__* values (*ε_r_*_,0*V*_ − *ε_E_ap,max__*), as compared to the *ε_r_*_,0*V*_ value. The highest tunability obtained at 1450 kV/cm is 94.6%. Meanwhile, the *FOM* reaches the maximum value (~37.6) at *E_ap,max_* of approximately 1000 kV/cm (Figure 6d). The reason for this behavior can be derived from the dielectric loss in Figure 6b and from the tunability in Figure 6c, in which the tunability increases gradually with *E_ap,max_*, but the dielectric loss increases slowly in the *E_ap,max_*-range of 300–1000 kV/cm and then rapidly with higher *E_ap,max_* values. On the other hand, the largest *FOM* (~37.6) is achieved at *E_ap,max_* of 1000 kV/cm, due to the optimum balance in tunability (88.7%) and low dielectric loss (0.0236).

## 4. Conclusions

In conclusion, the dependence of dielectric and tunability properties and of the *FOM* of the relaxor-ferroelectric PLZT films on film thickness, frequency and applied electric field were investigated. Comparatively, thicker films have satisfactory dielectric constants, tunability and *FOM* values, which can be attributed to the existence of a thin layer with lower dielectric constant near the interface between PLZT film and SRO electrode. A slight decrease in dielectric constant, tunability and *FOM* at high frequencies is caused by the oscillation of free dipoles in an alternating applied electric field. Moreover, the dielectric constant and tunability increase with increasing applied electric field. The 1000 nm PLZT film exhibits a high tunability of 94.6% at a maximum applied electric field of 1450 kV/cm at 100 kHz. Meanwhile, due to the combination of low dielectric loss (0.0236) and optimum tunability (88.7%) at a maximum applied electric field of 1000 kV/cm and 100 kHz, the largest *FOM* (37.6) is achieved in the 1000 nm PLZT film, making it highly promising candidate for integration into dielectric microwave tunable devices.

## Data Availability

Not applicable.

## Supplementary Materials

The following are available online at https://www.mdpi.com/article/10.3390/ma14216448/s1, Figure S1: (a) XRD theta-2theta around the PLZT(002)/(200) peaks. (b) Variation in the unit-cell volume and (c) change in ratio between the intensity of (002) and (200) peaks and ratio between out-of-plane and in-plane (*c*/*a*), of the PLZT films with changing film thickness; Figure S2: (a) Polarization hysteresis (*P*-*E*) loops and (b) corresponding switching current (*I_SW_-E*) curves of PLZT films with various film thicknesses, measured at 800 kV/cm and 1 kHz. (c) *P-E* loops and (d) corresponding *I_SW_-E* curves of 1000-nm PLZT film, measured at 1 kHz and under different applied electric fields (400, 800 and 1800 kV/cm); Figure S3: (a) Dielectric constant versus temperature dependence at various frequencies. Zoom in (b) low-field region of Figure 2c, (c) high-field region of Figure 2c and (d) high-field region of Figure 2d; Figure S4: (a) Dependence of dielectric constant at zero-bias field (*ε*_*r*,0*V*_) of 1000-nm film at *E_ap,max_* of 800 kV/cm on frequency. (b) Inverse capacitance density (*A*/*C_eff_*) and *ε*_*r*,0*V*_ value as a function of PLZT film thickness; Figure S5: (a) Normalized dielectric constant at zero field (*ε*_*r*,0*V*_) and the difference between *ε*_*r*,0*V*_ and *ε_E_ap,max__*, (dielectric at certain bias field) as a function of frequency. (b) Normalized tunability (*T*) and figure-of-merit (*FOM*) as a function of frequency. The measurements were done at 800 kV/cm for 1000-nm PLZT films; Figure S6: (a) Polarization hysteresis (*P-E*) loop and switching current curve, measured at 200 kV/cm and 1 kHz; (b) Dielectric constant curves measured at 1 kHz and various maximum applied electric fields (*E_ap,max_* of 250 and 500 kV/cm), of normal-ferroelectric Pb(Zr_0.52_Ti_0.48_)O_3_ films.
