# Supplementary material for: Relaxor-Ferroelectric Films for Dielectric Tunable Applications: Effect of Film Thickness and Applied Electric Field
Source: Materials (Basel). 2021 Oct 27;14(21):6448. doi: 10.3390/ma14216448 (PMC8585368; doi:10.3390/ma14216448)
Supplement: Supplementary file 1 [file materials-14-06448-s001.zip › materials-1421962-supplementary.pdf]

Relaxor-Ferroelectric Films for Dielectric Tunable Applications: Effect of Film Thickness and Applied Electric Field

Minh D. Nguyen [1,2,*], Doan T. Tran [2], Ha T. Dang [2,3], Chi T. Q. Nguyen [2,3], Guus Rijnders [1] and Hung N. Vu [2,*]

[1] MESA+ Institute for Nanotechnology, University of Twente, P.O. Box 217, 7500AE Enschede,
The Netherlands; a.j.h.m.rijnders@utwente.nl

[2] International Training Institute for Materials Science (ITIMS), Hanoi University of Science and Technology, 1 Dai Co Viet Road, Hanoi 100000, Vietnam; doantranthi97@gmail.com (D.T.T.); danghaktck@gmail.com (H.T.D.); chintq@vnuf.edu.vn (C.T.Q.N.)

[3] Mechanics and Civil Engineering, Vietnam National University of Forestry, Chuong My district, Hanoi 100000, Vietnam

* Correspondence: d.m.nguyen@utwente.nl (M.D.N.); hung.vungoc@hust.edu.vn (H.N.V.)

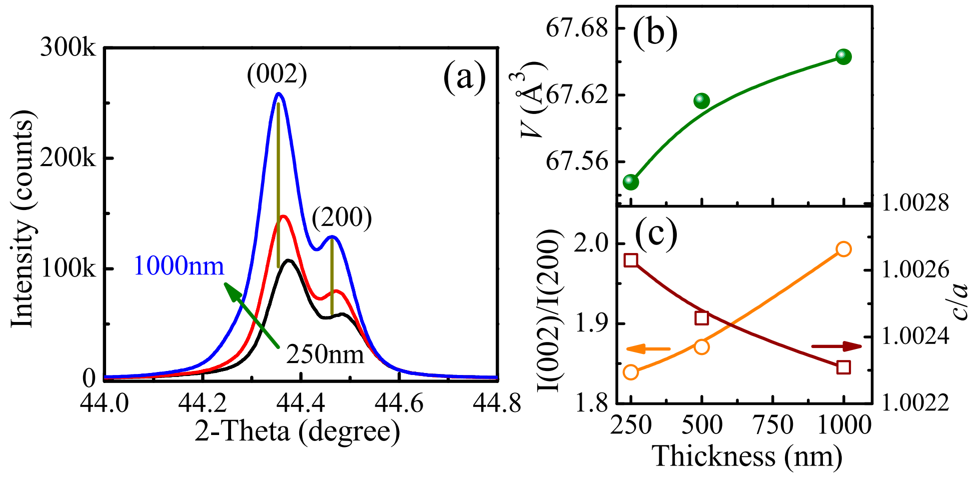

Figure S1. (**a**) XRD theta-2theta around the PLZT(002)/(200) peaks. (**b**) Variation in the unit-cell volume and (**c**) change in ratio between the intensity of (002) and (200) peaks and ratio between out-of-plane and in-plane (*c*/*a*), of the PLZT films with changing film thickness.

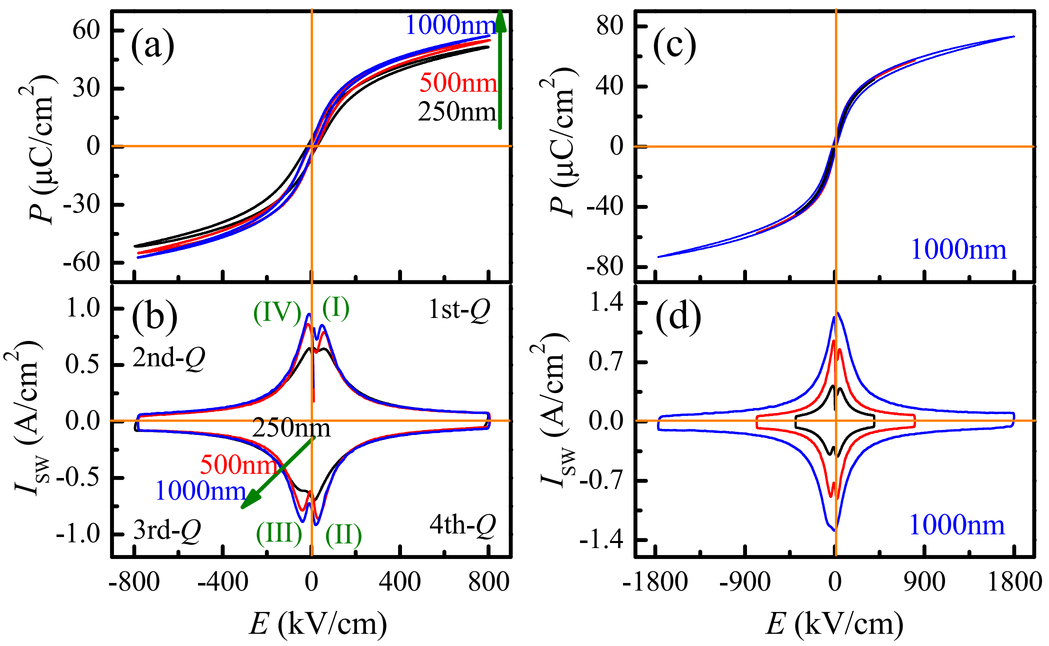

Figure S2. (**a**) Polarization hysteresis (*P*−*E*) loops and (**b**) corresponding switching current (I_{SW}−*E*) curves of PLZT films with various film thicknesses, measured at 800 kV/cm and 1 kHz. (**c**) *P*−*E* loops and (**d**) corresponding I_{SW}−*E* curves of 1000−nm PLZT film, measured at 1 kHz and under different applied electric fields (400, 800 and 1800 kV/cm). *Q* is denoted for the quadrant (e.g., 1st-*Q* is the first quadrant).

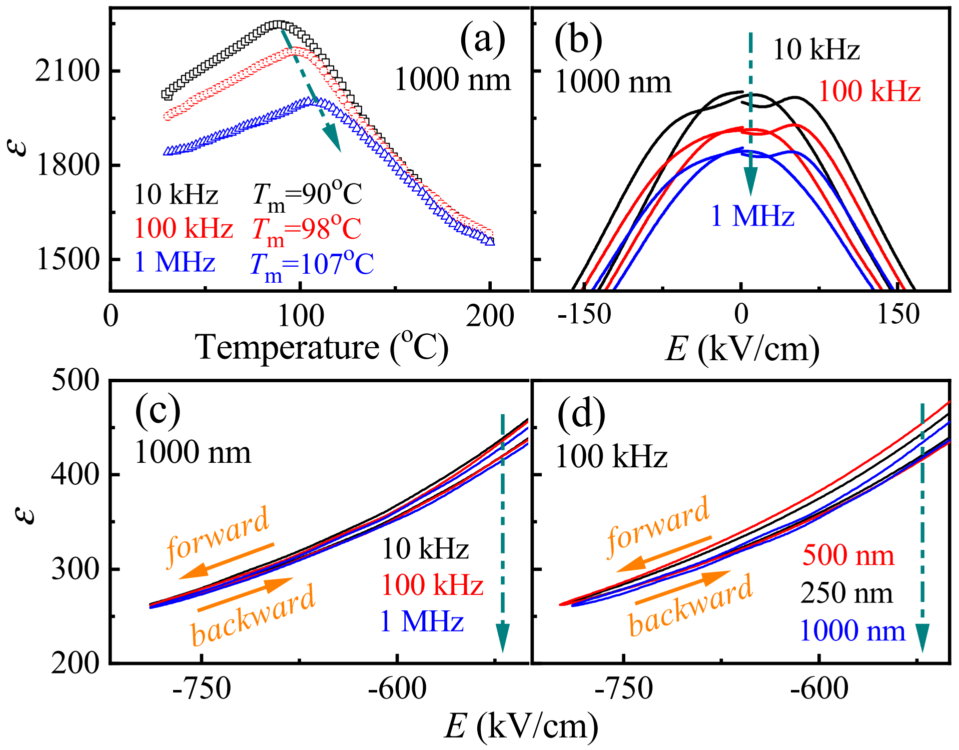

Figure S3. (**a**) Dielectric constant versus temperature dependence at various frequencies. Zoom in (**b**) low-field region of Fig. 2c, (**c**) high-field region of Fig. 2c and (**d**) high-field region of Fig. 2d.

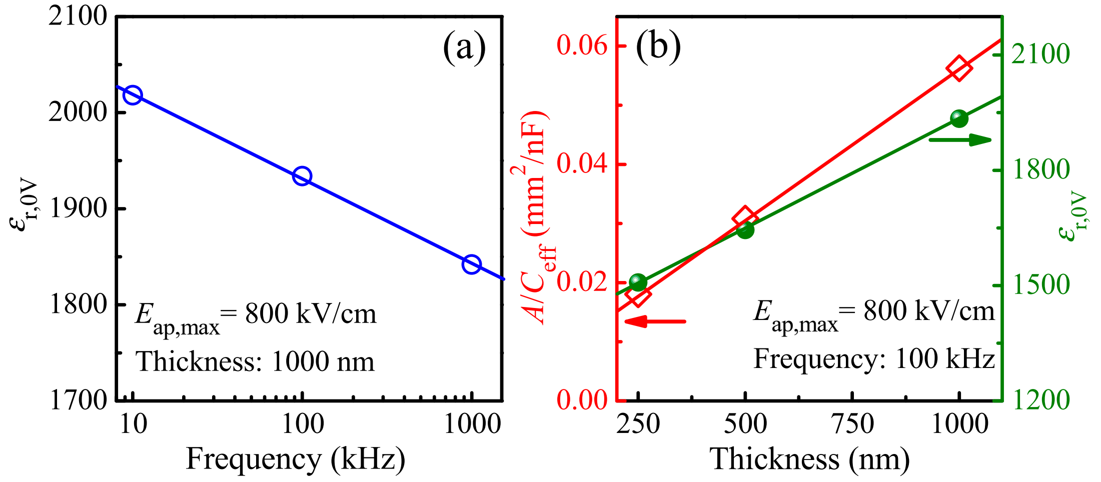

Figure S4. (**a**) Dependence of dielectric constant at zero-bias field ($\varepsilon_{r,0V}$) of 1000–nm film at $E_{ap,max}$ of 800 kV/cm on frequency. (**b**) Inverse capacitance density (A/C_{eff}) and $\varepsilon_{r,0V}$ value as a function of PLZT film thickness.

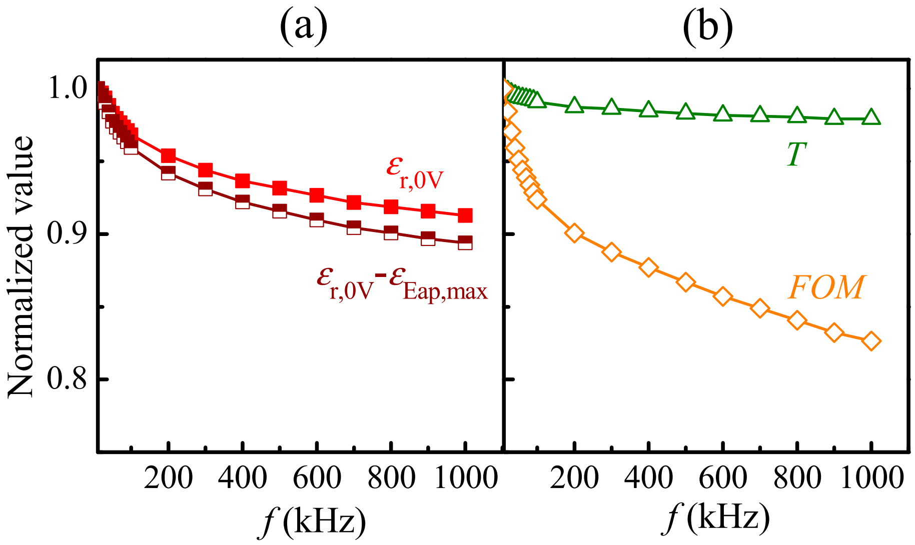

Figure S5. (**a**) Normalized dielectric constant at zero field ($\varepsilon_{r,0V}$) and the difference between $\varepsilon_{r,0V}$ and $\varepsilon_{Eap,max}$, (dielectric at certain bias field) as a function of frequency. (**b**) Normalized tunability (*T*) and figure-of-merit (*FOM*) as a function of frequency. The measurements were done at 800 kV/cm for 1000-nm PLZT films.

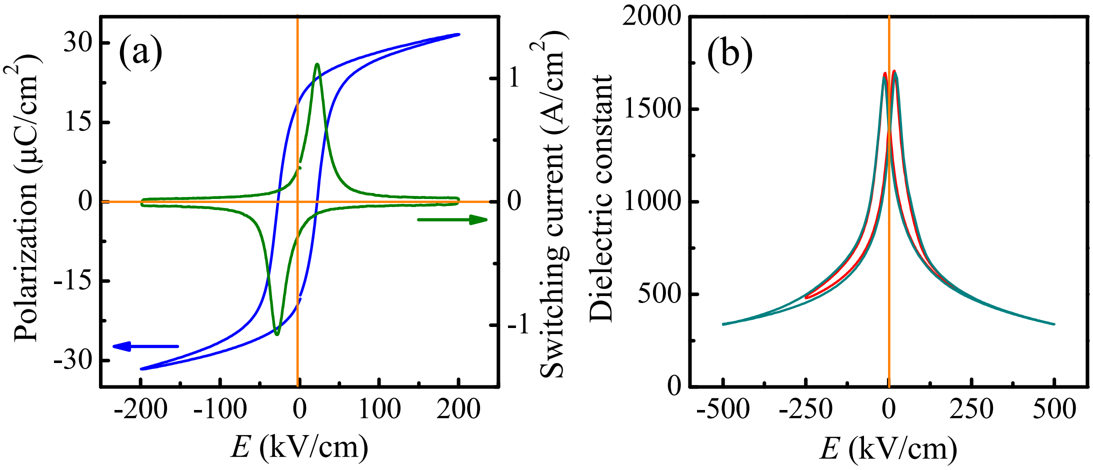

Figure S6. (**a**) Polarization hysteresis (*P*−*E*) loop and switching current curve, measured at 200 kV/cm and 1 kHz; (**b**) Dielectric constant curves measured at 1 kHz and various maximum applied electric fields ($E_{ap,max}$ of 250 and 500 kV/cm), of normal-ferroelectric $Pb(Zr_{0.52}Ti_{0.48})O_3$ films.
